# Editorial: Translating Innovations in Stroke Rehabilitation to Improve Recovery and Quality of Life Across the Globe

**DOI:** 10.3389/fneur.2020.630830

**Published:** 2020-12-14

**Authors:** Mayowa O. Owolabi, Thomas Platz, David Good, Bruce H. Dobkin, Echezona N. D. Ekechukwu, Leonard Li

**Affiliations:** ^1^Department of Medicine, College of Medicine, University of Ibadan, Ibadan, Nigeria; ^2^University College Hospital Ibadan, Ibadan, Nigeria; ^3^Blossom Specialist Medical Center, Ibadan, Nigeria; ^4^BDH-Klinik Greifswald, Institute for Neurorehabilitation and Evidence-Based Practice, University of Greifswald, Greifswald, Germany; ^5^Neurorehabilitation Research Group, Universitätsmedizin Greifswald, Greifswald, Germany; ^6^Department of Neurology, Pennsylvania State University, Philadelphia, PA, United States; ^7^Neurologic Rehabilitation and Research Program, Susan and David Wilstein Geffen School of Medicine, University of California, Los Angeles, Los Angeles, CA, United States; ^8^Department of Medical Rehabilitation, Faculty of Health Sciences, College of Medicine, University of Nigeria, Enugu, Nigeria; ^9^Environmental and Occupational Health Unit, College of Medicine, Institute of Public Health, University of Nigeria, Enugu, Nigeria; ^10^LANCET Physiotherapy, Wellness and Research Centre, Enugu, Nigeria; ^11^Division of Rehabilitation, Department of Medicine of Tung Wah Hospital Hong Kong, Hong Kong, Hong Kong

**Keywords:** stroke rehabilitation, constant therapy, low and mid income countries, robot therapy, diffusion tension imaging (DTI), stroke outcome

Stroke is a leading cause of neurological disability (1) and the second leading cause of death worldwide (2). Stroke incidence and burden is unevenly distributed globally. More than four-fifths of stroke mortality occurs in low and middle income countries (LMICs) (3), while the stroke disability adjusted life years (DALYs) is about seven times higher in LMICs than high income countries (HICs) (4). Also, stroke appears to occur more frequently in younger populations in LMICs compared to HICs (5). Generally, stroke-survivors have impaired function (6), activity limitations (7) participation restrictions (8), and a concomitantly reduced quality of life (9). Therefore, the primary goals of stroke rehabilitation (SR) are to enhance functional recovery and improve post-stroke quality of life.

A fundamental concept that underlies most SR-programmes is neuroplasticity (10). It is the process of reorganizing the structures, connections and function of the (central) nervous system in relation to some extrinsic and/or intrinsic stimulus (11). Conventional or traditional stroke rehabilitation relative to the innovative SR-programmes are less technology-driven and were in the past, broadly categorized into the “neurophysiological” (e.g., neurodevelopmental therapy, NDT/Bobath) and task-oriented approaches (TOA) (12). However, most therapy strategies now seem to use an impairment-oriented approach (e.g., arm basis training, mirror therapy, arm rehabilitation robot therapy, neuromuscular electrical stimulation etc.) when motor control is too impaired to be able to practice a real-world task, while TOA practice (e.g., task-specific arm rehabilitation, constraint induced movement therapy, body weight-supported treadmill training) can follow when motor control permits (13, 14). Even these interventions, however, may at times be no better than structured practice with goal-setting and feedback.

Currently, SR is also moving in directions enabled by advancements in technology and neurobiological research. Several invasive, but still experimental interventions include deploying stem cells or neural precursor cells and training with implanted electrodes to drive or read a circuit, which can create a brain-machine interface. Potential non-invasive adjunct innovations for practice include virtual reality, robotics, transcranial direct current stimulation, repetitive transcranial magnetic stimulation, etc.

There is a paucity of evidence from controlled trials that any one of these high technology rehabilitation techniques can provide clinically important improvements beyond present conventional treatments. For example, the Robot Assisted Training for the Upper Limb after Stroke (RATULS) study (15) did not reveal better gains compared to conventional therapy, despite its initial promise. These technologically complex strategies are also not likely to be feasible for LMIC populations, unless the results of practical RCTs reveal robust results.

In addition, the COVID-19 pandemic has led to greater use of tele-medicine (tele-rehabilitation). Over the Internet, the type, quantity, and quality of practice in the home/community can be monitored remotely and feedback provided by a therapist in brief weekly sessions to optimize training frequency, intensity, and progression (16). Wearable sensors that detect movement, instrumented and virtual reality devices that detect movement and touch, and video games or instructions that can monitor practice strategies for skill acquisition, strengthening, and conditioning may offer low-cost rehabilitation interventions for those who cannot afford the cost or travel time to attend an outpatient clinic (17). This is a very promising path for stroke rehabilitation in LMIC regions that could be supported by remotely based expertise in HICs.

Major limitations in the application of the most innovative high-technology SR-techniques (especially in the LMICs) include problems of acceptability, availability, affordability, and accessibility. Most innovative techniques are not only very expensive, but also require rigorous training and certification despite being adjunct therapies. However, a major advantage of some of the innovative techniques includes their ability to deliver high doses of therapy, minimize manual patient handling and its concomitant occupational hazards, their automated feedback mechanisms, and their higher reliability that makes them quite suitable for research purposes.

The contributing articles to this Research Topic elaborated further on translating some innovations in SR to clinical practice. In a retrospective study that compared the effects of home-based and clinic-based Constant Therapy on aphasia, Godlove et al., reported similar treatment outcomes in both groups, although home-based users had significantly higher treatment frequency and mastery rate than the clinic-based users. Platz systematically reviewed literature on SR-guidelines and reported that the available guidelines have a national focus that reflected on the healthcare situations in HICs. He therefore recommended the development of international SR-guidelines with greater focus on feasible, evidence-based therapies.

In order to effectively translate the innovations of robot therapy for SR, Duret et al., in an overview of robot assisted therapy for stroke-survivors, proposed a two-step approach: (1) a robot therapy focused on impairment and (2) a therapist-based translation of the gains to functional activities. Ekechukwu et al., in a systematic review of pragmatic solutions for stroke recovery in LMICs, found that most of the studies with innovatively high technology interventions were conducted in HICs. They did, however, report several traditional and low-cost interventions that significantly contributed to recovery and improvement of quality of life of stroke-survivors (Ekechukwu et al.).

Apart from innovative interventions, it is also important to consider innovative tools for predicting stroke outcome and assessing self-perceived burden of disability. Moura et al., in a narrative review of diffusion tensor imaging (DTI) biomarkers for predicting post-stroke motor outcomes reported a lack of consensus on a gold-standard for DTI biomarkers. They did, however, report a significant correlation between DTI biomarkers and motor impairments and thereafter recommended studies that will determine the predictive values of DTI biomarkers for motor disability models. Also, Wei et al., in their prospective study of stroke-survivors reported a high degree of self-perceived burden and recommended interventions to address this problem.

In conclusion, LMICs settings with abysmally low ratios of neurorehabilitation professionals to population may 1 day benefit from the availability and utilization of some innovative high-technology techniques, once they have been shown to produce better outcomes for particular impairments. Cellular and pharmacological interventions for neural repair are being tried, often based on rather modest animal model outcome assessments, so they are still far from pointing to any clinically important reduction in specific impairments. The principles for best internationally applicable training strategies, whether for the LMIC or HIC, may not differ as much as expected in regard to best practices. International rehabilitation guidelines that emphasize commonalities and ways to surmount barriers may better serve all communities (18).

## Author Contributions

This editorial was conceived by MOO. First draft of the manuscript was developed by MOO and ENDE. The manuscript was edited and approved by all authors.

## Conflict of Interest

The authors declare that the research was conducted in the absence of any commercial or financial relationships that could be construed as a potential conflict of interest.
